# Impact of UGT1A1*28 Allele on the Safety and Effectiveness of Sacituzumab Govitecan in Metastatic Triple-Negative Breast Cancer: Real-World Evidence

**DOI:** 10.3390/jcm15020574

**Published:** 2026-01-10

**Authors:** Fernando do Pazo-Oubiña, Betel del Rosario García, Marta Miarons, Eva M. Legido Perdices, Elena Prado Mel, Ruth Ramos Díaz, Fernando Gutiérrez Nicolás, on behalf of the Estudio Mama-SSG Working Group

**Affiliations:** 1 Pharmacy Department, Son Espases University Hospital, Cra. de Valldemossa, 79, 07120 Palma, Spain; nandodo_pazo@hotmail.com; 2 eHealth and Telemedicine Research Group, Health Research Institute of the Balearic Islands (IdISBa), Cra. Valldemossa, 07120 Palma, Spain; 3 Unidad de Investigación, Servicio de Farmacia Hospitalaria, Complejo Hospitalario Universitario de Canarias, 38320 Santa Cruz de Tenerife, Spain; 4 Pharmacy Department, Consorci Hospitalari de Vic, 08500 Barcelona, Spain; 5 Pharmacy Department, Vall d’Hebron Hospital Universitari, Vall d’Hebron Barcelona Hospital Campus, 08035 Barcelona, Spain; 6 Servicio de Farmacia Hospitalaria, Hospital Arnau de Vilanova-Llíria, 46160 Valencia, Spain; 7 Servicio de Farmacia Hospitalaria, Hospital Universitario Virgen del Rocío, 41013 Sevilla, Spain; 8 Instituto de Investigación Sanitaria de Canarias (IISC), 35012 Canarias, Spain

**Keywords:** germline mutations, *UGT1A1*, Sacituzumab govitecan, triple negative breast cancer, drug toxicity, febrile neutropenia

## Abstract

**Background:** The *UGT1A1* gene is associated with the toxicity caused by SN38, the cytotoxic component of Sacituzumab govitecan (SG) used in the treatment of metastatic triple-negative breast cancer (mTNBC), among other approved indications. In this study, we aimed to analyze the effect of UGT1A1*28 allele on the safety and, secondarily, the effectiveness of SG in mTNBC. **Methods:** This was a multicenter, ambispective study that included patients treated with SG for mTNBC. Genotyping for UGT1A1*28 was performed using real-time polymerase chain reaction (PCR). Adverse events (AEs) of grade ≥ 2 during the first three cycles were compared between patients who were homozygous mutant (UGT1A1*28/*28) and those with wild-type (WT) or heterozygous genotypes. Effectiveness between the two groups was also compared using progression-free survival (PFS) and overall survival (OS) assessed with the Kaplan–Meier method. **Results:** A total of 81 patients were included: 37.0% were WT, 55.6% heterozygous, and 7.4% homozygous mutant. All UGT1A1 *28/*28 patients experienced grade ≥ 2 AEs (100% vs. 69.3%; *p* = 0.109), with a statistically significant association in the case of febrile neutropenia (33.3% vs. 6.7%; *p* = 0.025), and a trend towards higher rates of anemia and diarrhea (50.0% vs. 17.3%; *p* = 0.053). Genotype did not influence PFS or OS; however, dose reductions were associated with better survival outcomes. **Conclusions:** This real-world study shows a correlation between toxicity and the presence of the UGT1A1*28 mutation in patients treated with SG for mTNBC. Improving treatment tolerability through dose reductions may enhance SG effectiveness. These findings support the implementation of *UGT1A1* genotyping in routine clinical practice.

## 1. Introduction

Triple-negative breast cancer (TNBC) accounts for approximately 15–20% of all breast cancers cases and is characterized by the absence of estrogen receptors, progesterone receptors, and lack of human epidermal growth factor receptor 2 (HER2) overexpression. This subtype exhibits aggressive biological behavior, a high risk of recurrence and poor prognosis [1].

In metastatic triple-negative breast cancer (mTNBC), treatment options are limited due to the absence of molecular targets. As a result, conventional chemotherapy remains the cornerstone of systemic treatment, except in patients with increased PD-L1 expression or pathogenic BRCA1/BRCA2 variants [2]. With conventional chemotherapy, the median progression-free survival (PFS) for previously treated mTNBC is only 2–3 months, with a median overall survival (OS) of approximately 8–15 months [3]. These poor outcomes, which reflect the high risk of early relapse and short survival in this population, highlight the clinical need for new therapeutic strategies.

Sacituzumab govitecan (SG), an antibody-drug conjugate targeting trophoblast cell surface antigen 2 (Trop-2), was approved by the U.S. Food and Drug Administration (FDA) in 2020 for the treatment of mTNBC in patients who have received at least two prior systemic therapies, including one for metastatic disease [4,5].

SG consists of a humanized monoclonal antibody linked to SN-38, the active metabolite of irinotecan, and has demonstrated superior efficacy compared to chemotherapy in the ASCENT trial, with a median PFS of 5.6 months versus 1.7 months (hazard ratio [HR] 0.4; 95% confidence interval [CI] 0.32–0.52; *p* < 0.001) and a median OS of 12.1 versus 6.7 months (HR 0.48; 95% CI 0.38–0.59; *p* < 0.001) [6]. Upon binding to Trop-2 on tumor cells, the conjugate is internalized, releasing SN-38 intracellularly. This compound inhibits topoisomerase I, causing DNA damage and subsequent tumor cell apoptosis. Nevertheless, the cytotoxic component of SG (SN-38) is associated with adverse effects (AEs) such as neutropenia and diarrhea, also observed with irinotecan [7].

The metabolism of SN-38 is mediated by uridine-diphosphoglucuronosyl transferase 1A1 (*UGT1A1*), an enzyme responsible for glucuronidation [8]. This process converts SN-38 into SN-38 glucuronide, an inactive and water-soluble metabolite that facilitates biliary and renal excretion. Genetic polymorphisms in the *UGT1A1* gene, particularly the *28 allele, can result in reduced enzymatic activity and individuals homozygous for this variant (*28/*28) are at increased risk for toxicity when exposed to SN-38. Evidence from irinotecan studies suggests a clear association between the *28/*28 genotype and a higher incidence of grade ≥3 neutropenia and diarrhea [9,10]. Similar findings have been reported with liposomal irinotecan, for which an initial dose reduction is recommended in the Summary of Product Characteristics for certain indications [11].

Preliminary post hoc analyses from the ASCENT trial suggested that patients with the *28/*28 genotype may also experience higher rates of hematologic and gastrointestinal toxicities with SG. Furthermore, the proportion of patients requiring dose reductions for these AEs was significantly higher among women with the *28/*28 genotype [3].

Given these findings, the role of UGT1A1*28 as a potential predictive biomarker of toxicity in patients receiving SG deserves further evaluation in real-world clinical settings.

The objective of this study is to assess the association between UGT1A1*28 polymorphism and both the safety and effectiveness of SG in patients with mTNBC in routine clinical practice conditions.

## 2. Materials and Methods

### 2.1. Study Design

This was an observational, descriptive, nationwide, and ambispective study designed to evaluate the allelic frequency of UGT1A1*28 allele among patients diagnosed with mTNBC treated with SG. The study also aimed to assess the safety and effectiveness of SG in routine clinical practice. Recruitment was conducted from June 2023 to October 2024 across 13 centers in Spain.

The study adhered to Good Clinical Practice (GCP) guidelines, the Declaration of Helsinki, and applicable local and regulatory requirements. Patients were treated according to routine clinical practice, and the prescription of SG, including dosage and schedule, was independent of study participation. The study protocol was approved by the central institutional review board (IRB) of the Hospital Universitario de Canarias, Canarias, Spain. Written informed consent was obtained from all participants before any study-related procedures were conducted and before any genetic determination was carried out, the patient received an explanation of the study with an information sheet detailing the study objectives, procedures, and potential risks associated with genetic testing. Participation was entirely voluntary, and patients could withdraw at any time without consequences for their medical care.

### 2.2. Study Population

Patients eligible for the study were required to meet the following criteria: (1) Histologically or cytologically confirmed diagnosis of mTNBC; (2) aged ≥18 years; (3) scheduled to initiate or already receiving SG as part of standard care; and (4) able to provide written informed consent for participation, including genetic testing. Exclusion criteria included: (1) patients receiving SG as part of a clinical trial; (2) treated with SG for indications other than mTNBC; (3) initiation of treatment with a reduced dose and (4) refusal to provide consent for study participation or genetic testing.

### 2.3. Data Collection

Data were systematically collected using the REDCap electronic data capture system (hosted by the Spanish Society of Hospital Pharmacy), ensuring standardization and completeness. The following categories of information were included: Baseline data, which included demographics (age, sex, weight, height, and race), performance status (Eastern Cooperative Oncology Group [ECOG]) and prior lines of therapy (including neoadjuvant, adjuvant, and metastatic settings). Treatment and follow-up data covered treatment protocols, including initial SG dosing, modifications, and schedules, adverse events, supportive care and laboratory data during the first three treatment cycles.

### 2.4. Treatment Protocol

Patients received SG according to its approved clinical indication, at a dose of 10 mg/kg administered intravenously on days 1 and 8 of each 21-day cycle. Dose modifications, interruptions, or discontinuations were implemented at the treating physician’s discretion, based on observed toxicities. Dose reductions were categorized as 25% or 50% reductions from the initial dose.

### 2.5. Genetic Analysis

A digital lancet was used to collect the samples, obtaining a drop of blood (approximately 5 µL), which was deposited on a collection card (WhatmanTM 903^®^, GE Healthcare Bio-Sciences Corp., Chicago, IL, USA). A 3 mm diameter disk of dried blood was then punched from this card to obtain gDNA (genomic deoxyribonucleic acid). The sample underwent alkaline lysis (NaOH 0.2 M + Tris-HCl 40 mM + EDTA 0.55 mM) followed by centrifugation.

After gDNA extraction, genotyping focused on the *UGT1A1* promoter (TA)_n polymorphism, a short tandem repeat (STR) located in the regulatory region of the *UGT1A1* gene (dbSNP: rs3064744). This polymorphism consists of a variable number of (TA) dinucleotide repeats, with the (TA)_6 allele (or UGT1A1*1) being the most prevalent reference allele in the general population and the (TA)_7 allele, also known as UGT1A1*28, representing the most frequent reduced-function variant. Therefore, genotyping assays were performed using real-time polymerase chain reaction (PCR) with specific primers and a pair of fluorescent hybridization probes complementary to the mutant and reference alleles on a LightCycler^®^ 480 thermocycler. The oligonucleotides used (sense 5′–3′) were: CACCTTCTTTATCTCTGAAAGTGA (forward primer), GGGAACAGCCAGACAAAAG (reverse primer), FAM-CGATACACCAAGTTAATGTTTGACTGTGT-P (anchor probe) and CCTCTCCTACTTATATATATATATATATGGCAAAAACC-Cy5 (sensor probe), where FAM and Cy5 are fluorophores and P indicates a 3′-phosphate modification to prevent extension.

*UGT1A1* alleles were classified as: wild type (WT): *1/*1; heterozygous: *1/*28; and homozygous: *28/*28. The accuracy and reliability of genotyping were validated by including control samples of known genotypes in each assay batch.

### 2.6. Safety and Effectiveness Assessment

As previously stated, the objective was to assess frequency of UGT1A1*28 allele and the association between the UGT1A1*28 polymorphism and both the safety and effectiveness of SG in patients with mTNBC treated under routine clinical practice conditions.

To evaluate safety, adverse events (AEs) occurring during the first three cycles of treatment (six doses) were collected from clinical records and classified according to their severity using the National Cancer Institute Common Terminology Criteria for Adverse Events (NCI-CTCAE) version 5.0. Special attention was given to hematological toxicities (neutropenia, febrile neutropenia, and thrombocytopenia), gastrointestinal toxicities (diarrhea, nausea, and vomiting), and changes in liver or renal function parameters. The use of supportive treatments such as granulocyte colony-stimulating factors (G-CSFs), antiemetics, and atropine was recorded. Laboratory data included liver function tests (AST, ALT, and bilirubin) and creatinine levels at baseline and subsequent cycles. Hospital admissions related to AEs, dose interruptions, and permanent discontinuations of SG were also documented.

To assess effectiveness, PFS and OS were evaluated. PFS was defined as the time from the first dose of SG to documented disease progression or death from any cause; and OS was defined as the time from the first dose of SG to death from any cause.

### 2.7. Statistical Analysis

To evaluate the association between UGT1A1*28 genotypes and toxicity to SG, a Chi-Square test was performed to estimate the statistical significance between homozygous mutant patients and WT or heterozygous patients in terms of AEs rates, hospital admission due to AEs and rate of dose reduction or treatment discontinuation due to AEs.

For the analysis of PFS and OS of mTNBC patients treated with SG, a Kaplan–Meier analysis was performed. The Log-rank test was used to determine the statistical significance between the homozygous mutant patients and the rest of the participants.

Statistical analyses were performed using the statistical packages STATA 15 and IBM SPSS^®^ Statistics 25, with statistical significance defined as *p* < 0.05.

Additionally, a post hoc analysis (not pre-specified in the study protocol) was conducted to generate hypotheses regarding the potential influence of dose modification on treatment outcomes. The study population was stratified into four groups according to UGT1A1*28 genotype (WT + HET vs. HOM) and whether a dose reduction was required during treatment. Kaplan–Meier survival analyses and Log-rank tests were performed to compare PFS between these subgroups.

## 3. Results

### 3.1. Baseline Characteristics of the Included Population

**Figure 1:**
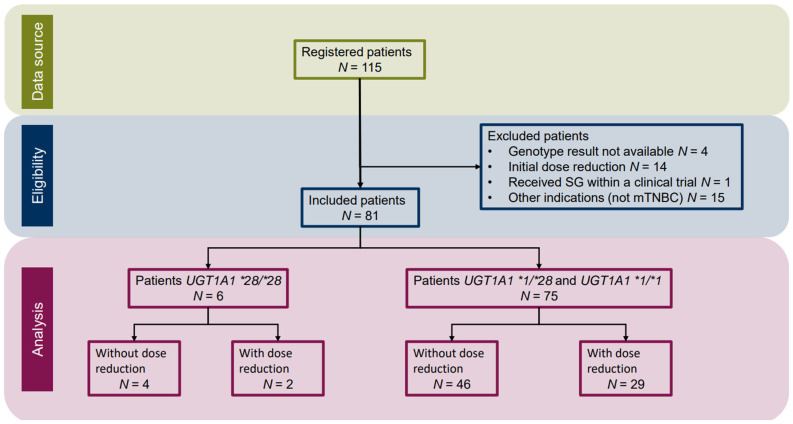
Patients’ flowchart. SG: Sacituzumab govitecan. mTNBC: Metastatic triple-negative breast cancer.

From an initial cohort of 115 patients, 81 women with mTNBC were included in the analysis. Patients were excluded because they did not have a mTNBC indication, were receiving SG within a clinical trial, initiated treatment with dose reductions, or lacked UGT1A1*28 genotyping (Figure 1).

Except for one patient, the final study population was Caucasian and had a mean age [range] of 54.5 years [31–78]. All patients had an ECOG performance status ≤ 1, and 95% received SG after at least two prior lines of therapy for metastatic disease.

The patients received a median of 12 doses of SG [range 1 to >30], with a median treatment duration of 6.2 months [range 3.1–18.7 months]. Data on race, previous adjuvant/neoadjuvant treatment, antiemetic prophylaxis, use of G-CSF and atropine prior to SG administration are summarized in Table 1.

Pharmacogenetic analysis revealed that 7.4% (*N* = 6) of the included patients were homozygous for the UGT1A1*28/*28 variant. Baseline characteristics of WT and heterozygous (WT + HET) patients were comparable to those of homozygous mutated (HomoMut) patients (Table 1).

### 3.2. Relationship Between SG Safety and Genotype in UGT1A1

**Figure 2:**
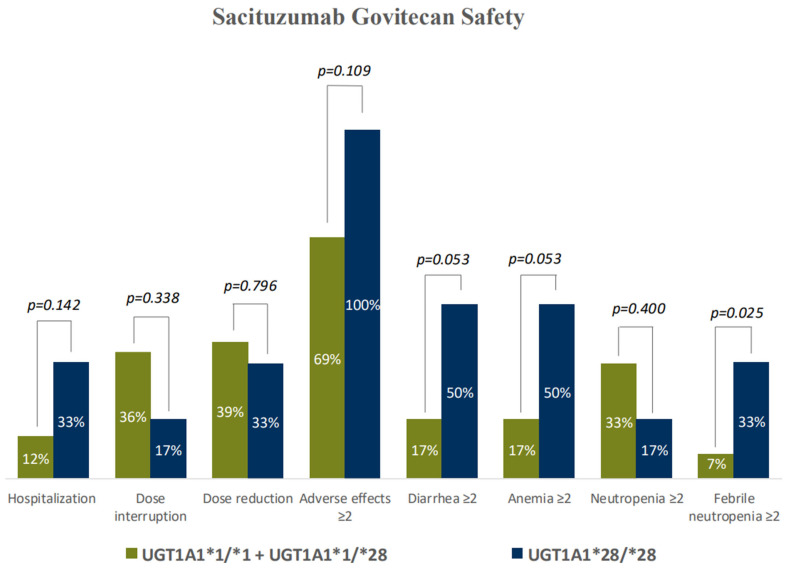
Rates of Sacituzumab-Govitecan toxicities according to the genotypes in *UGT1A1*.

The analysis of SG-related toxicity stratified by UGT1A1*28 genotype showed that 100% of HomoMut patients developed grade ≥ 2 AEs during the first three cycles, compared to 69% of WT + HET genotype patients (*p* = 0.109) (Figure 2). No significant differences were observed in the incidence of grade ≥ 2 neutropenia (HomoMut 17% versus WT + HET 33%; *p* = 0.400). However, the incidence of grade ≥ 2 febrile neutropenia was significantly higher in the HomoMut genotype group compared to the rest of the patients (33% versus 7%; *p* = 0.025).

Dose interruptions due to neutropenia or febrile neutropenia occurred in 24% and 17% of WT + HET and HomoMut patients respectively (*p* = 0.683), while dose reductions for the same reasons occurred in 19% and 17% of patients (*p* = 0.903), respectively. Importantly, in no cases did neutropenia or febrile neutropenia result in permanent discontinuation of SG treatment.

Additionally, there was a trend towards a higher rate of diarrhea in the HomoMut group (50% versus 17%; *p* = 0.053), as well as a similar trend for anemia (50% versus 17%; *p* = 0.053) (Figure 2).

There were no dose interruptions due to diarrhea in HomoMut patients, whereas 12% of WT + HET patients required interruption (*p* = 0.368). Dose reduction due to grade ≥ 2 diarrhea was required in 33% of HomoMut women and 11% of WT + HET women (*p* = 0.104). Regarding anemia, 9% of WT + HET patients interrupted doses due to this AE, while it was 17% in those HomoMut (*p* = 0.562). Eight percent of WT + HET patients and 17% of HomoMut patients required dose reduction due to grade ≥ 2 anemia. In neither group did anemia or diarrhea lead to permanent treatment discontinuation.

Regarding laboratory data (AST, ALT, bilirubin and creatinine), no significant differences were observed between HomoMut and WT + HET patients.

### 3.3. Relationship Between SG Effectiveness and UGT1A1 Genotype

**Figure 3:**
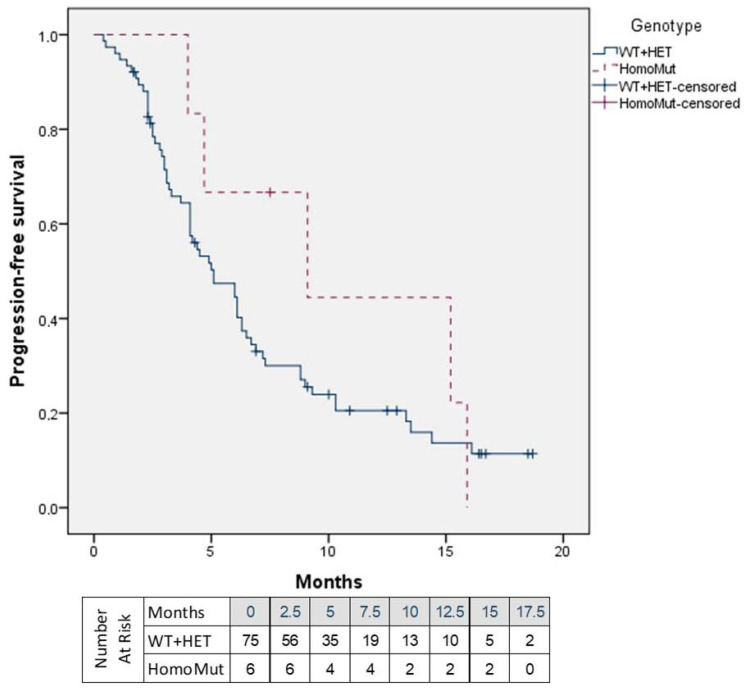
Progression-free survival according to *UGT1A1* genotype. HomoMut: UGT1A1*28/*28 genotype; WT + HET: UGT1A1*1/*1 and UGT1A1*1/*28 genotype.

**Figure 4:**
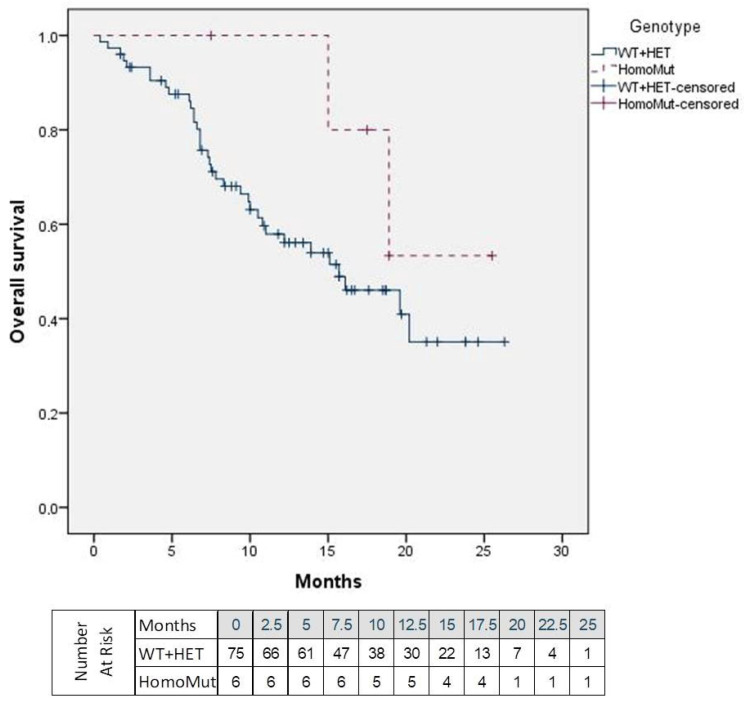
Overall survival according to *UGT1A1* genotype. HomoMut: Group of patients with UGT1A1*28/*28 genotype; WT + HET: Group of patients with UGT1A1*1/*1 and UGT1A1*1/*28 genotype.

To evaluate the potential impact of mutation in the *UGT1A1* gene on SG effectiveness, an assessment of PFS and OS was performed (Figure 3 and Figure 4). After a median follow-up of 9.8 months [range: 4.0 to 26.3], the median PFS was 9.1 months in the HomoMut group compared to 5.0 months in the WT + HET group (*p* = 0.311). The rate of progression-free patients was 20% (*N* = 15) in the WT + HET group and 17% (*N* = 1) in the HomoMut.

Regarding OS, the median was not reached in HomoMut patients, whereas it was 15.1 months in the WT + HET (*p* = 0.255). The proportion of patients alive at the end of follow-up was 66.7% (*N* = 4) in the HomoMut group and 53.3% (*N* = 40) in the WT + HET group.

### 3.4. Relationship Between SG Effectiveness and Dose Reduction

As a post hoc analysis to explore whether dose reduction might influence SG effectiveness, the population was stratified according to genotype and the need for dose reduction. When the four resulting groups were compared, differences were observed only in PFS among patients with WT + HET genotype (Table 2). Specifically, in the WT + HET group, the median PFS was 4.1 months for patients receiving the full dose and 6.9 months for those who underwent dose reduction (*p* = 0.015).

**Figure 5:**
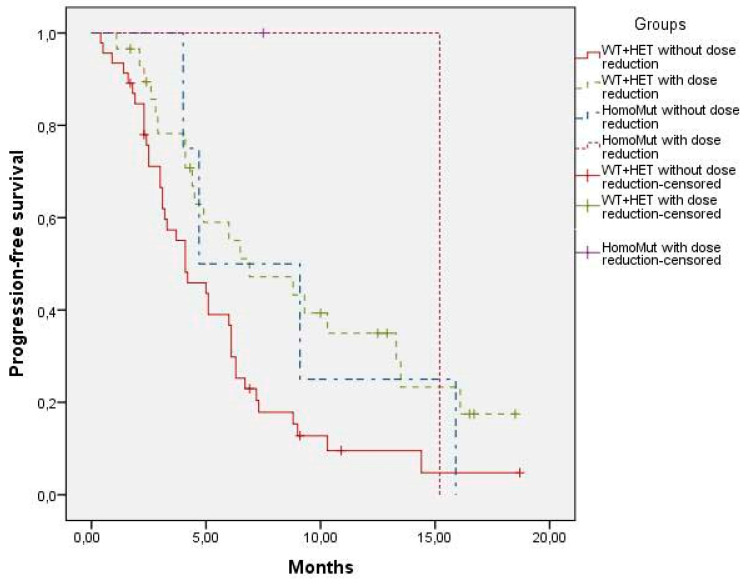
Progression-free survival according to *UGT1A1* genotype and dose reduction. HomoMut: UGT1A1*28/*28 genotype; WT + HET: UGT1A1*1/*1 genotype and UGT1A1*1/*28 genotype. WT + HET Without dose reduction (*N* = 46), WT + HET With dose reduction (*N* = 29), HomoMut Without dose reduction (*N* = 4), HomoMut With dose reduction (*N* = 2).

Among HomoMut patients, the median PFS was 9.1 months for those receiving the full dose and 15.2 months for those requiring dose reduction; however, this difference did not reach statistical significance (*p* = 0.782). (Figure 5 and Table 2)

## 4. Discussion

The present study showed a 7.4% frequency of UGT1A1*28/*28 genotype among patients diagnosed with mTNBC and treated with SG under routine clinical practice conditions. Although no statistically significant differences were found (the sample size did not allow these differences to be identified), 100% of patients with the HomoMut genotype experienced grade ≥ 2 adverse events during the first three cycles, compared to 69% of patients with the WT + HET genotype (*p* = 0.109).

Regarding effectiveness, no significant differences were found between groups; however, a trend towards better outcomes was noted in HomoMut patients, with a median PFS of 9.1 months versus 5.0 months, and median OS not reached versus 15.1 months for HomoMut and WT + HET patients.

Except for the study conducted by Alaklabi et al. [12], which found no UGT1A1*28/*28 genotype among 115 patients with mTNBC, the frequency of UGT1A1*28/*28 genotype observed in our cohort was slightly lower than that reported in previous studies including predominantly Caucasian populations (13% in [2]), 25% in [13]) 12% in [14], and 9% in [15]). The primary factor that may account for the lower observed frequency of homozygotes is the exclusion of patients who initiated treatment with a reduced dose. Notably, three of these 14 patients were HomoMut. If these cases were included in the analysis, the frequency of the UGT1A1*28/*28 genotype would increase to 9.5%.

The rate of grade ≥ 2 febrile neutropenia was higher among HomoMut patients and, although no statistically significant differences were observed in the other AEs analyzed, there was a clear trend toward a greater toxicity in the HomoMut group, particularly in terms of grade ≥ 2 diarrhea and anemia. These findings may be confirmed in future studies with larger sample sizes. Previous studies, such as that by Rugo et al. [3], have already reported rates of severe febrile neutropenia, diarrhea and anemia approximately three times higher in HomoMut patients. We believe it is important to highlight that, while overall neutropenia rates seemed to be higher in WT + HET patients, the incidence of febrile neutropenia was higher among HomoMut patients. Although our sample size is limited, this observation has also been found in the study by Rugo et al. [3], where grade ≥ 3 neutropenia occurred in 50% vs. 59% of cases and grade ≥ 3 febrile neutropenia in 4% vs. 18% of cases for WT + HET vs. HomoMut patients, respectively. These findings suggest a greater severity of neutropenia among HomoMut patients. It is also worth noting that none of the participants in our study required permanent treatment discontinuation due to toxicity, supporting the overall safety of SG when administered with appropriate pharmacotherapeutic monitoring.

Regarding effectiveness, we also found no statistically significant difference in median PFS and median OS between HomoMut and WT + HET patients. We must emphasize that our study was not designed to demonstrate differences in terms of effectiveness. Furthermore, the small sample size, particularly in the HomoMut group (n = 6), means that our results should be interpreted with caution due to the lack of statistical power to detect a difference. Taking all this into account, our findings are consistent with a recent systematic review and meta-analysis, which included eleven studies and over 1500 patients, and reported no significant differences, with pooled median PFS of 4.9 months and OS of 9.6 months, respectively [16]. In addition, our data on median PFS and OS among WT + HET patients are comparable to the results reported by Alaklabi et al. [12]. Other similar studies did not provide data on median PFS or OS according to *UGT1A1* mutational status.

As regards the need for dose adjustment due to toxicity, it is well established that dose reductions in SG are considerably more frequent in women homozygous for the UGT1A1*28 mutation (35%) compared to those who are WT or heterozygous (18% and 19%, respectively) [3]. Despite this, clinical response in patients treated with SG does not appear to be compromised by dose reductions or interruptions, as efficacy outcomes were similar to those observed in patients receiving the full drug dose. In our cohort, however, we found a similar rate of dose reduction between HomoMut and WT + HET patients. Therefore, to better understand the potential impact of dose adjustment on SG effectiveness and to generate hypothesis, we analyzed survival outcomes in relation to dose reduction in each group of patients.

While acknowledging the limitations of this post hoc analysis and the small sample size, no genotype-related differences in PFS were identified (Figure 3). However, WT + HET patients who underwent dose reduction demonstrated significantly longer PFS than those receiving the full SG dose (Figure 5, Table 2). In the HomoMut group, a similar trend was observed, although it did not reach statistical significance, possibly due to the small sample size (Figure 5, Table 2).

These findings suggest that dose reductions, far from compromising effectiveness, may be associated with better toxicity control and potentially improved survival, as previously described by Rugo et al. [3], who reported that patients requiring dose reductions due to toxicity experienced longer PFS compared to those receiving the full drug dose (8.3 vs. 4.6 months).

In line with our findings, a recent real-world retrospective study also evaluated the association between the UGT1A1*28 polymorphism and both toxicity and treatment outcomes in 68 patients with mTNBC treated with SG [13]. Interestingly, that study reported a higher frequency of *28/*28 homozygosity (25%) compared to our cohort (7.4%). Despite this difference, they similarly observed that patients homozygous for the *28 allele experienced a significantly greater rate of treatment discontinuation due to toxicity (HR 5.52; *p* = 0.03), although no differences were found in terms of disease progression. These results reinforce the notion that *UGT1A1* genotyping may help identify patients at increased risk of early toxicity, while not necessarily predicting worse oncologic outcomes. Moreover, as in our cohort, the authors highlighted that with appropriate dose adjustments and supportive care, the treatment remains feasible even in genetically predisposed patients.

Our study has several limitations. First, the relatively small sample size, and, particularly, the low proportion of patients with the UGT1A1*28/*28 genotype, limited the statistical power to identify significant differences or draw firm conclusions regarding toxicity or effectiveness across subgroups. Second, part of the data was collected retrospectively from medical records, which may have led to variability in the completeness and accuracy of adverse event reporting depending on clinician practices and timing of documentation. Third, the absence of a standardized protocol for dose modifications and supportive care across participating centers could have introduced heterogeneity in management, potentially influencing outcomes. Lastly, as an observational study, causal relationships cannot be established, and residual confounding cannot be excluded.

Despite these limitations, our work provides valuable real-world data on the safety and effectiveness of SG according to *UGT1A1* genotypes. With nationwide coverage and information collected from 81 patients treated in daily clinical practice, this study contributes to the external validity of current evidence and highlights the need for prospective studies with larger, more balanced cohorts.

## 5. Conclusions

In summary, our findings suggest that UGT1A1*28/*28 is a predictive genetic marker of SG-related toxicity. Although a recent consensus on the management of SG toxicity in mTNBC mentions that it is difficult to determine the clinical utility of *UGT1A1* [17], the presence of this genotype is associated with a higher frequency of adverse events, particularly febrile neutropenia. Therefore, as some authors recommend [18,19], initial dose reductions in this drug in homozygous mutated patients may improve health outcomes, not only in terms of safety and quality of life, but also survival. These results support the implementation of routine *UGT1A1* genotyping to personalize SG treatment in clinical practice, similar to the established use of *DPYD* (dihydropyrimidine dehydrogenase) genotyping prior the administration of fluoropyrimidines in colorectal cancer.

## Figures and Tables

**Table 1:** Baseline characteristics of the study population (N = 81).

		WT + HET (*N* = 75)	HomoMut (*N* = 6)	*p*-Value
Average age, years [IQR]		54.8 [31–78]	53.8 [47–73]	0.828
Race	Caucasian, *N* (%)	74 (98.7)	6 (100)	0.776
	Asian (%), *N* (%)	1 (1.3)	0 (0)	
BMI, kg/m^2^ [IQR]		26.1 [15.9–35.6]	27.8 [21.1–44.6]	0.254
Patients who received neoadjuvant/adjuvant therapy, *N* (%)		54 (72)	4 (67)	0.780
SG as ≥2 line of treatment for metastatic disease, *N* (%)		72 (96)	5 (83)	0.733
Average treatment time with SG, months [IQR]		5.9 [3.1–18.7]	9.4 [4.0–15.9]	0.082
Use of antiemetic prophylaxis	3-drug combination (5-HT3 receptor antagonists + corticosteroids + neurokinin-1 (NK-1) receptor antagonists), *N* (%)	68 (91)	6 (100)	0.433
	2-drug combination (5-HT3 receptor antagonists + corticosteroids), *N* (%)	7 (9)	0 (0)	
Use of atropine prior to SG administration, *N* (%)		8 (11)	0 (0)	0.399
Primary prophylaxis with G-CSF, *N* (%)		33 (44)	2 (33)	0.611
Secondary prophylaxis with G-CSF, *N* (%)		19 (25)	2 (33)	0.667

5-HT3: serotonin 5-hydroxytryptamine receptor type 3; BMI: Body Mass Index; G-CSF: granulocyte colony-stimulating factors; HomoMut: homozygous mutated; IQR: Interquartile range; NK-1: neurokinin-1; SG: Sacituzumab govitecan; WT + HET: wild type and heterozygous.

**Table 2:** Statistical analysis (Log Rank test with pairwise comparisons) to study the differences between the median progression-free survival (months) (*p*-Value).

Genotypes	WT + HET Without Dose Reduction (*N* = 46)	WT + HET with Dose Reduction (*N* = 29)	HomoMut Without Dose Reduction (*N* = 4)	HomoMut with Dose Reduction (*N* = 2)
WT + HET Without dose reduction (*N* = 46)		4.1 versus 6.9 (0.015)	4.1 versus 9.1 (0.225)	4.1 versus 15.2 (0.121)
WT + HET With dose reduction (*N* = 29)	6.9 versus 4.1 (0.015)		6,9 versus 9.1 (0.972)	6,9 versus 15.2 (0.445)
HomoMut Without dose reduction (*N* = 4)	9.1 versus 4.1 (0.225)	9.1 versus 6.9 (0.972)		9.1 versus 15.2 (0.782)
HomoMut With dose reduction (*N* = 2)	15.2 versus 4.1 (0.121)	15.2 versus 6.9 (0.445)	15.2 versus 9.1 (0.782)	

HET: heterozygous; HomoMut: homozygous mutated; WT: Wild type.

## Data Availability

The datasets generated during and/or analyzed during the current study are available from the corresponding author on reasonable request.

## References

[1] Foulkes W.D., Smith I.E., Reis-Filho J.S. (2010). Triple-negative breast cancer. N. Engl. J. Med..

[2] Gennari A., André F., Barrios C.H., Cortés J., de Azambuja E., DeMichele A., Dent R., Fenlon D., Fenlon J., Hurvitz S.A. (2021). ESMO Clinical Practice Guideline for the diagnosis, staging and treatment of patients with metastatic breast cancer. Ann. Oncol..

[3] Rugo H.S., Tolaney S.M., Loirat D., Punie K., Bardia A., Hurvitz S.A., O’Shaughnessy J., Cortés J., Diéras V., Carey L.A. (2022). Safety analyses from the phase 3 ASCENT trial of sacituzumab govitecan in metastatic triple-negative breast cancer. NPJ Breast Cancer.

[4] Zaman S., Jadid H., Denson A.C., Gray J.E. (2019). Targeting Trop-2 in solid tumors: Future prospects. OncoTargets Ther..

[5] Syed Y.Y. (2020). Sacituzumab Govitecan: First Approval. Drugs.

[6] Bardia A., Hurvitz S.A., Tolaney S.M., Loirat D., Punie K., Oliveira M., Brufsky A., Sardesai S.D., Kalinsky K., Zelnak A.B. (2021). Sacituzumab Govitecan in Metastatic Triple-Negative Breast Cancer. N. Engl. J. Med..

[7] Goldenberg D.M., Cardillo T.M., Govindan S.V., Rossi E.A., Sharkey R.M. (2015). Trop-2 is a novel target for solid cancer therapy with sacituzumab govitecan (IMMU-132), an antibody-drug conjugate (ADC). Oncotarget.

[8] Immunomedics, Inc. (2023). Trodelvy (Sacituzumab-Govitecan-HZIY) [Package Insert]. U.S. Food and Drug Administration Website. https://www.accessdata.fda.gov/drugsatfda_docs/label/2023/761115s035lbl.pdf.

[9] Hu Z.Y., Yu Q., Pei Q., Guo C. (2010). Dose-dependent association between UGT1A1*28 genotype and irinotecan-induced neutropenia: Low doses also increase risk. Clin. Cancer Res..

[10] Liu X., Cheng D., Kuang Q., Liu G., Xu W. (2014). Association of UGT1A1*28 polymorphisms with irinotecan-induced toxicities in colorectal cancer: A meta-analysis in Caucasians. Pharmacogenomics J..

[11] Les Laboratoires Servier Industrie (2024). Onivyde Pegylated Liposomal (Irinotecan Hydrochloride Trihydrate) [Package Insert]. European Medicines Agency Website. https://www.ema.europa.eu/en/documents/product-information/onivyde-pegylated-liposomal-epar-product-information_en.pdf.

[12] Alaklabi S., Roy A.M., Zagami P., Chakraborty A., Held N., Elijah J., George A., Attwood K., Shaikh S.S., Chaudhary L.N. (2025). Real-World Clinical Outcomes With Sacituzumab Govitecan in Metastatic Triple-Negative Breast Cancer. JCO Oncol. Pract..

[13] Wong M.H., Jones V.C., Yu W., Bosserman L.D., Lavasani S.M., Patel N., Sedrak M.S., Stewart D.B., Waisman J.R., Yuan Y. (2024). UGT1A1*28 polymorphism and the risk of toxicity and disease progression in patients with breast cancer receiving sacituzumab govitecan. Cancer Med..

[14] Loriot Y., Petrylak D.P., Rezazadeh Kalebasty A., Fléchon A., Jain R.K., Gupta S., Bupathi M., Beuzeboc P., Palmbos P., Balar A.V. (2024). TROPHY-U-01, a phase II open-label study of sacituzumab govitecan in patients with metastatic urothelial carcinoma progressing after platinum-based chemotherapy and checkpoint inhibitors: Updated safety and efficacy outcomes. Ann. Oncol..

[15] Heong V.Y.M., Marmé F., Rugo H.S., Tolaney S.M., Bardia A., Schmid P., Verret W., Valdez T., Wang H., Cortes J. (2023). Safety outcomes by UGT1A1 status in the phase III TROPiCS- 02 study of sacituzumab govitecan (SG) in HR+/HER2e metastatic breast cancer (mBC). Ann. Oncol..

[16] Sultana R., Chen S., Lim E.H., Dent R., Chowbay B. (2024). Efficacy and safety of sacituzumab govitecan Trop-2-targeted antibody-drug conjugate in solid tumors and UGT1A1*28 polymorphism: A systematic review and meta-analysis. BJC Rep..

[17] Valsecchi A.A., Pisegna S., Antonuzzo A., Arpino G., Bianchini G., Biganzoli L., Colloca G.F., Criscitiello C., Danesi R., Fabi A. (2025). Delphi consensus on the management of adverse events in patients with metastatic triple-negative breast cancer treated with sacituzumab govitecan. Oncol..

[18] Karas S., Innocenti F. (2022). All You Need to Know About UGT1A1 Genetic Testing for Patients Treated With Irinotecan: A Practitioner-Friendly Guide. JCO Oncol. Pract..

[19] Legido Perdices E.M., do Pazo Oubiña F., Prado Mel E., Miarons M., García B.D.R., Gutiérrez Nicolás F. (2025). Influence of the UGT1A1 gene polymorphism on treatment with sacituzumab govitecan. Narrative review. Farm. Hosp..

